# The Role of Preprocedural Computed Tomography Angiography in Enhancing Arterial Embolisation for Life-Threatening Haemoptysis: A Case Series

**DOI:** 10.3390/arm93060057

**Published:** 2025-12-11

**Authors:** Anna Ziętarska, Adam Dobek, Piotr Białek, Wojciech Szubert, Sebastian Majewski, Ludomir Stefańczyk

**Affiliations:** 1 Department of Radiology and Diagnostic Imaging, Norbert Barlicki Memorial Teaching Hospital No. 1, Medical University of Lodz, 90-153 Lodz, Poland; 2 Department of Pneumology, Norbert Barlicki Memorial Teaching Hospital No. 1, Medical University of Lodz, 90-153 Lodz, Poland

**Keywords:** haemoptysis, artery embolisation, computed tomography angiography, non-bronchial systemic arteries, interventional radiology, cystic fibrosis

## Abstract

**Highlights:**

**What are the main findings?**

- Although bronchial arteries are the most common source of haemoptysis, a wide range of systemic arteries—from the subclavian to the phrenic—may contribute to the development of pathological vascular supply.
- Computed tomography angiography (CTA) performed from the neck to the L2 vertebral level, with a wide imaging field, enables precise localisation of the culprit bleeding site and more accurate identification of systemic collaterals.

**What is the implication of the main finding?**

- Due to the dynamic nature of pulmonary vascular remodelling, wide-field CTA should be included in the qualification process for every subsequent embolisation procedure rather than being limited to the initial intervention.
- Performing CTA with a wide imaging window covering all potential feeders arising from the thoracic aorta increases the likelihood of detecting collateral vessels, thereby improving procedural planning and potentially enhancing clinical outcomes in patients at particular risk of non-bronchial systemic artery involvement.

**Abstract:**

Haemoptysis arises from diverse respiratory diseases and may involve a broad spectrum of thoracic vessels. Arterial embolisation (AE) is an effective, repeatable, minimally invasive treatment option for life-threatening haemoptysis. This case series included 10 patients (mean age 34 years; six males; five with cystic fibrosis) who underwent 17 AE procedures for life-threatening haemoptysis between January 2018 and September 2025. The study assessed the role of wide-field computed tomography angiography (CTA), extending from the thoracic inlet to L2, in preprocedural planning, bleeding localisation and detection of systemic collaterals. CTA accurately predicted the culprit region in 16 out of 17 procedures. Non-bronchial systemic arteries were identified in 6 out of 10 patients, consistent with previous reports. CTA showed strong concordance with angiography and enabled the detection of uncommon collaterals, including subclavian and phrenic branches. Recurrence of hemoptysis occurred in one patient during follow-up; however, three patients were lost to follow-up. Wide-field CTA enhances the identification of systemic feeders and supports procedural planning, potentially reducing recurrence associated with missed culprit vessels. AE remains a valuable option for haemoptysis control in cystic fibrosis, with outcomes further improved following initiation of CFTR modulators. The small sample size and incomplete follow-up limit generalisability, but findings highlight the importance of CTA in guiding AE and improving clinical outcomes.

## 1. Introduction

Haemoptysis, defined as the expectoration of blood or blood-streaked sputum originating from the lower respiratory tract, may result from a wide spectrum of thoracic pathologies. The most common causes include infections such as tuberculosis (TB) and aspergillosis, malignancies, bronchiectasis and cystic fibrosis (CF) [1,2].

Symptoms may range from mild blood-streaking of sputum to the expectoration of larger volumes of fresh blood. Although haemoptysis is often self-limited, 5–15% of episodes are potentially life-threatening, primarily due to asphyxiation but also haemorrhagic shock [1,2,3,4]. There is no universally accepted volume threshold defining massive, life-threatening or non-massive haemoptysis. Definitions vary across studies, relying either on estimated expectorated volume or clinical context, such as respiratory compromise or haemodynamic instability. Recurrent haemoptysis is also common, negatively affecting quality of life regardless of its severity. It may reflect progression of the underlying disease or failure to control the culprit vessel during previous treatment [5].

Embolisation is a minimally invasive procedure that involves the endovascular injection of embolic material via a catheter. This results in permanent or temporary vessel occlusion, depending on the embolic agent used.

Artery embolisation (AE) is a well-established technique for the management of haemoptysis and has a wide spectrum of applications across diverse pulmonary conditions. It may serve as a palliative therapy by reducing blood loss and improving patient comfort, for example, in patients with lung cancer who are not candidates for surgical resection due to poor performance status [6,7]. AE also functions as a life-saving interventional procedure in cases of massive haemoptysis [8]. Moreover, it can be performed repeatedly in the same patient, which is of particular importance in chronic diseases such as CF, where it may additionally act as a bridging therapy before lung transplantation [9,10].

Accurate targeting of the bleeding source enhances the clinical efficacy of AE and optimises preprocedural planning by the interventional radiologist [11,12].

This approach is essential for patients who have had multiple AEs. Occlusion of the artery and chronic inflammation may promote the development of collateral circulation arising from systemic vessels, most commonly the bronchial arteries (BAs), subclavian artery and its branches—particularly the internal mammary artery (IMA), as well as intercostal and phrenic arteries [13,14].

During patient selection for AE, computed tomography (CT) and bronchoscopy are commonly employed as standard diagnostic modalities.

Bronchoscopy helps identify the pulmonary segment that may contain the source of bleeding [15]. In certain situations, such as the need for immediate airway control, bronchoscopy is prioritised over CT [10]. However, due to the dynamic changes of the pulmonary vasculature, it is crucial for the interventionist to localise the origin of the culprit vessel as precisely as possible.

According to the guidelines of the American College of Radiology (ACR) and the Cardiovascular and Interventional Radiological Society of Europe (CIRSE), contrast-enhanced CT of the chest is considered the diagnostic modality of choice for preprocedural evaluation in patients with haemoptysis [10,16]. CT angiography (CTA) enhances the identification of the culprit vessel before AE. Identifying bleeding predictors on CT scans, such as ground-glass opacities (GGOs), consolidations, pleural thickening or artery diameter enlargement, provides precise guidance to the target site [10,17,18,19,20].

Considering the broad variability of systemic arteries as collateral feeders, CTA protocols should cover the entire thorax with a wide imaging window and extend from the neck to the L2 vertebrae, ensuring comprehensive visualisation of the thoracic aorta and its collateral pathways.

Compared with bronchoscopy, CT offers the additional advantage of depicting vascular anatomy, allowing the identification of critical anastomoses—such as spinal collaterals. This information assists in selecting appropriate embolic materials and helps prevent complications from non-target embolisation [10].

Omission of culprit vessels, particularly non-bronchial systemic arteries (NBSAs), remains a frequent cause of AE inefficiency [21,22]. Improved identification of bleeding sources may enhance procedural efficacy. Despite growing interest, further research is required to assess the clinical benefits of preprocedural imaging.

The aim of this case series was to evaluate the role of wide-field CTA in preprocedural planning and outcome assessment of AE in patients presenting with life-threatening haemoptysis.

## 2. Materials and Methods

### 2.1. Study Design and Patient Population

We conducted a single-centre, retrospective case series analysis including patients who underwent AE for massive haemoptysis at Norbert Barlicki University Teaching Hospital No. 1 in Lodz between January 2018 and September 2025. Inclusion criteria required that all patients had undergone CTA prior to embolisation.

Anonymised patient data were obtained from hospital records and imaging archives. Since no identifiable information was accessed and no interventions were performed, Ethics Committee review was waived.

### 2.2. Procedures

#### 2.2.1. CTA

Contrast-enhanced chest CTA was performed in all patients as part of the preprocedural qualification for AE, using a Revolution CT scanner (GE Healthcare, Milwaukee, WI, USA).

Preprocedural CTA was performed with a broad imaging range—from the neck to the level of the L2 vertebra—covering the entire thoracic aorta and lungs, to ensure complete visualisation of potential bronchial and non-bronchial systemic feeders relevant to procedural planning.

The imaging protocol included a non-contrast overview scan followed by an arterial-phase acquisition, performed after intravenous administration of iodinated contrast medium Ultravist 370 (Bayer Healthcare, Berlin, Germany).

The primary aim of CTA was to identify the source of bleeding and to evaluate the underlying aetiology of haemoptysis. Preprocedural CTA reports were systematically reviewed to identify vascular abnormalities (e.g., bronchial artery dilatation, tortuosity, pleural or parenchymal changes) potentially associated with haemoptysis. Findings were then compared with the vessel embolised during AE to assess concordance. Post-procedural reassessment was performed with follow-up CTA where indicated.

#### 2.2.2. AE

A radiologist with specialist training performed all embolisation procedures. Vascular access was achieved via a common femoral artery puncture, followed by selective catheterisation of the suspected artery using a 5F catheter.

Digital subtraction angiography (DSA) was conducted to identify the culprit vessel and to exclude spinal artery supply. Embolisation was performed using coils, Glubran 2 (Gem S.r.l., Viareggio, Italy) with Lipidol mixture, microspheres or PHIL polymer (MicroVention Inc., Aliso Viejo, CA, USA), selected according to vessel anatomy and operator preference. In one case, the embolic material used was not documented.

### 2.3. Analysis of Culprit Vessel Distribution and Comparative Review with Prior Studies

We analysed the distribution of culprit vessels identified in our study population, categorising them into BAs and NBSAs (including intercostal, internal thoracic, inferior phrenic and subclavian arteries). For contextualisation, our findings were compared with previously published large series that reported abnormal vessel distribution [11,12,23,24]. The frequency of each vessel type was summarised in figures.

## 3. Results

**Figure 1:**
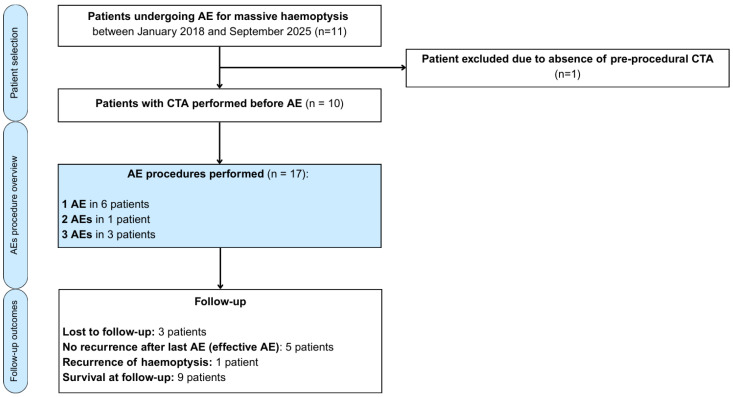
Flow diagram illustrating patient selection, number of arterial embolisation procedures performed and follow-up outcomes in patients with massive haemoptysis.

Among 11 patients who underwent AE during the study period, 1 was excluded due to the absence of preprocedural CTA (Figure 1). In total, 17 AE procedures were performed in 10 patients, revealing 15 BAs and 7 NBSAs as the culprit sources of bleeding.

The mean age and standard deviation (SD) of the study population was 34± 14 years (range: 19–62), with CF being the predominant aetiology, present in five patients. Among the 10 patients included, 6 were males, and 4 were females. The most frequently used embolic materials were coils and glue, each applied in six procedures.

Baseline patient characteristics are summarised in Table 1.

**Figure 2:**
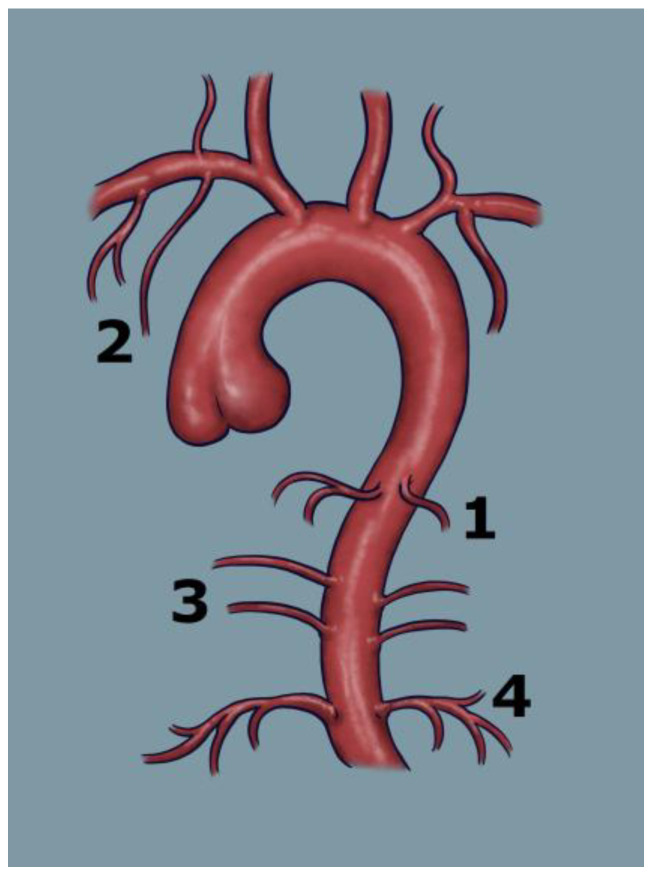
Schematic illustration of the aorta and its main branches contributing to the pulmonary circulation, which may serve as potential targets for embolisation: 1—bronchial arteries, 2—subclavian artery, 3—intercostal arteries, 4—phrenic arteries.

Figure 2 schematically illustrates the points of origin of the major systemic arteries arising from the aorta, highlighting branches that may give rise to collateral vessels and represent potential sources of bleeding.

Six patients (60.0%) demonstrated at least one bleeding NBSA identified during angiography. Patient characteristics with identified NBSAs are shown in Table 2. The mean number of targeted NBSAs per patient was 0.70 (SD = 0.67).

**Figure 3:**
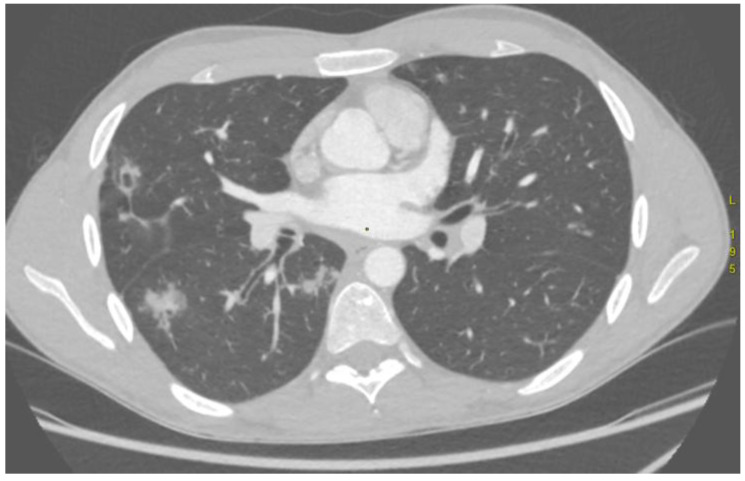
Computed tomography angiography demonstrates intrabronchial haemorrhage in the right lung, seen as hyperdense regions.

The outcomes of the comparison between CTA findings and the culprit vessel identified during DSA are presented in Table 3. In nearly all AE procedures (16/17), changes in pulmonary parenchyma, such as areas of consolidation indicative of alveolar haemorrhage (Figure 3), and thoracic vasculature, such as vessel diameter, tortuosity and anatomical configuration, corresponded with the bleeding source confirmed on DSA.

The DLP for wide-field CTA ranged from 176.53 to 884.67 mGy∙cm, adjusted to patient body habitus.

**Figure 4:**
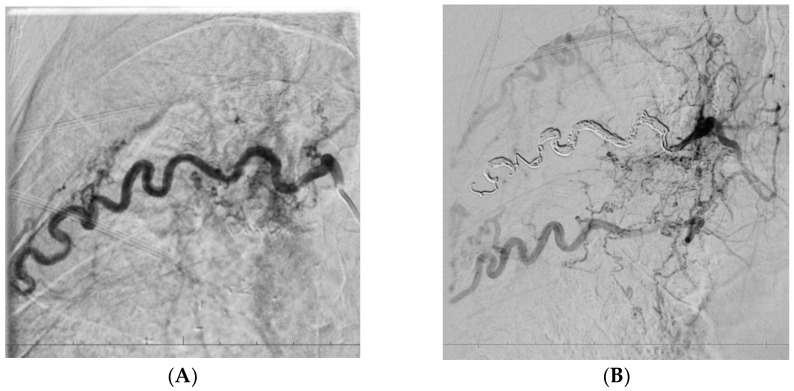
A 35-year-old patient after the third artery embolisation procedure for persistent haemoptysis secondary to aspergillosis, currently under evaluation for sarcoidosis. Embolisation of an enlarged right intercostal artery (**A**), supplying the affected region, resulted in altered pressure gradients within the adjacent vascular network and subsequent development of multiple collateral vessels (**B**).

**Figure 5:**
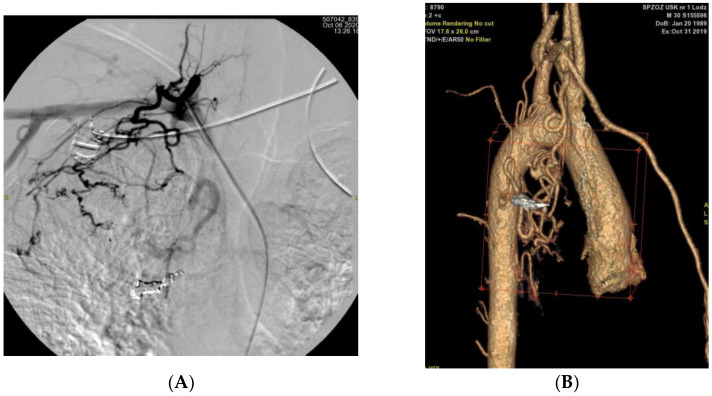
(**A**): Angiographic image obtained after bronchial artery embolisation, demonstrating the development of collateral vessels arising from the subclavian artery (**B**): Three-dimensional CT reconstruction of the aorta and its branches showing collateral formation originating from the subclavian arteries following bronchial artery embolisation.

In two patients who underwent follow-up imaging before planned re-embolisation, the development of collateral vessels supplying previously embolised lung regions was observed. These collaterals originated from the subclavian and intercostal arteries (Figure 4 and Figure 5).

In patient P02, post-procedural DSA performed immediately after embolisation of the right intercostal artery revealed early recruitment of collateral circulation (Figure 4). In contrast, in patient P01, collateral flow from the subclavian artery was identified 1 year after the first embolisation procedure (Figure 5).

In another patient who underwent two AE procedures for haemoptysis originating from the right BA territory, subsequent bleeding arose from the contralateral (left) lung, where the culprit vessel was identified as a branch of the subclavian artery.

## 4. Discussion

The present case series demonstrates the practical value of wide-field CTA in planning AE for life-threatening haemoptysis. CTA accurately identified the culprit region in 16 of 17 procedures and enabled reliable detection of NBSAs, including rare feeders such as subclavian and phrenic branches—providing clinically relevant guidance in patients with complex vascular anatomy. This was particularly evident in individuals with CF, whose disease profile predisposes them to recurrent bleeding, often arising from remote systemic vascular territories. In this subgroup, haemoptysis control was achieved following the combination of AE and CFTR modulator therapy. In one patient (P01), haemoptysis persisted despite three AE procedures and resolved after initiation of CFTR modulator therapy. Of the two additional CF patients who underwent AE and started CFTR modulators, one (P09) remained free of recurrence during follow-up (27 months), while the other (P07) did not experience recurrent haemoptysis but died 4 years after the single AE procedure due to spontaneous pneumothorax. Although the small number of cases limits firm conclusions, these observations highlight the potential complementary roles of AE and contemporary medical therapy. Taken together, these findings support the use of comprehensive CTA coverage (neck to L2) as an integral component of AE planning, particularly in patients with complex or evolving vascular profiles, where omission of systemic feeders may contribute to recurrence of haemoptysis.

BAs are the primary source of haemoptysis and the most frequent targets for embolisation, although bleeding may also originate from NBSAs [25]. Given the complexity of the thoracic vascular system and anatomical inter-individual variation, it is desirable to locate the bleeding site in advance. The AE procedure requires identification of the bleeding vessel part by contrast injection under continuous X-ray guidance. To optimise the radiation dose, contrast volume and procedure timing, preceding non-invasive methods—such as computed tomography or bronchoscopy—are crucial for narrowing the target area [10,26].

Our findings confirm the key role of CTA in localising the bleeding source before AE. In almost all cases (16/17; 94.1%), the preprocedural CT accurately predicted the affected lung and the region of bleeding, providing crucial guidance for the interventionist. This finding is comparable to the ranges reported in the literature. Li et al. reported 98.8% accuracy between MDCTA and DSA, while Le et al. 97.5% [11,24]. The strong agreement between preprocedural CTA findings and angiographic confirmation in almost all cases underscores the value of CTA in preprocedural assessment and planning.

Earlier studies demonstrated clear progress in the diagnostic capability of CTA for detecting NBSAs. In the work by Yoon et al., published in 2005, which was performed using a 16-detector-row CT scanner, NBSAs were correctly identified in 62% of cases compared with conventional angiography, while 100% of BAs were detected [27]. More than a decade later, Li et al., in 2018, conducted a study using a 256-row multidetector CT, which achieved nearly complete concordance with DSA, detecting 34 of 35 NBSAs in addition to all of the BAs [11].

Many authors highlight the role of NBSA detection in preventing recurrent haemoptysis [25,28]. Martin et al. emphasised that many re-embolisations come from NBSAs [14]. In our study population, NBSAs were identified in 6 out of 10 patients. In the literature, the prevalence has been reported to range from 36% to 75%, with the highest rates typically observed in studies conducted on cystic fibrosis populations [14,20,25].

Compared with the study by Li et al., in which the average number of culprit NBSAs per patient was 0.36 (SD = 0.84), our study population demonstrated a higher mean of 0.7 (SD = 0.67) per patient [11]. This discrepancy may reflect differences in study populations, as in our series, 5 of the 10 embolised patients had cystic fibrosis, which is consistent with the above statistics.

A widely accepted hypothesis regarding NBSA involvement in haemoptysis recurrence suggests that chronic inflammation promotes neovascularisation, particularly in patients with CF, aspergillosis or TB [29,30,31,32]. The newly formed vessels are fragile and susceptible to rupture, especially under the increased haemodynamic stress imposed by compensatory enlargement of adjacent arteries. In addition to the formation of new vessels, pre-existing collaterals may open shortly after embolisation as a result of increased intravascular pressure [33,34,35].

Embolisation in chronic conditions such as CF does not address the underlying disease—it is a symptomatic intervention aimed at stabilising the patient’s condition, which is particularly important for those awaiting lung transplantation. In one patient with a history of TB who presented in critical condition with haemoptysis, large high-flow systemic–pulmonary arterial shunts supplying the right pulmonary artery were identified, and AE maintained clinical stability until successful lung transplantation.

Significant benefit was also observed in patients in whom haemoptysis was controlled through AE and CFTR modulator therapy was subsequently initiated. In one patient who had undergone three AE procedures, haemoptysis ceased to recur only after the introduction of CFTR modulators.

The presence of NBSAs is an unfavourable prognostic factor in itself. However, failure to identify and embolise these vessels is a major contributor to recurrent haemoptysis. CTA acts as a protective factor in this context, as it increases the likelihood of proper identification of culprit vessels, even in patients with bilateral pathological lesions [17,30].

In their study, Martin et al. proposed a policy of performing CTA within 6 months before AE, based on their findings that NBSAs were identified in up to 75% of procedures in patients with CF [14]. Apart from contributing to recurrent haemoptysis, NBSAs may also serve as a source of massive haemoptysis [12,29]. Therefore, preprocedural CTA should extend beyond patients with chronic pulmonary disease and be incorporated into diagnostic algorithms for massive haemoptysis, as long as it does not delay urgent, life-saving intervention.

**Figure A1:**
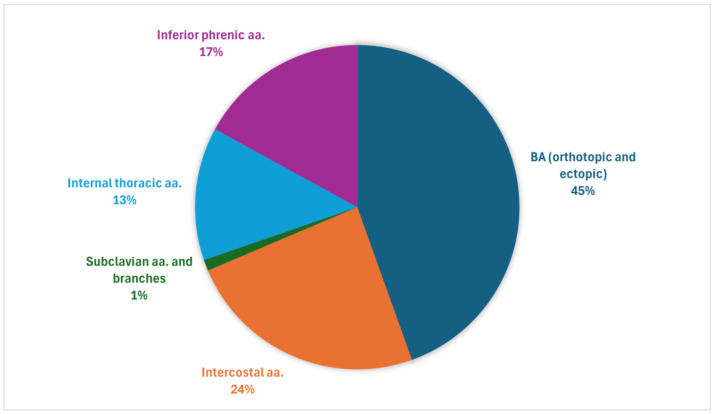
Overall distribution of haemoptysis-related arteries: aggregated data from our study population and four prior series [11,12,23,24].

**Figure A2:**
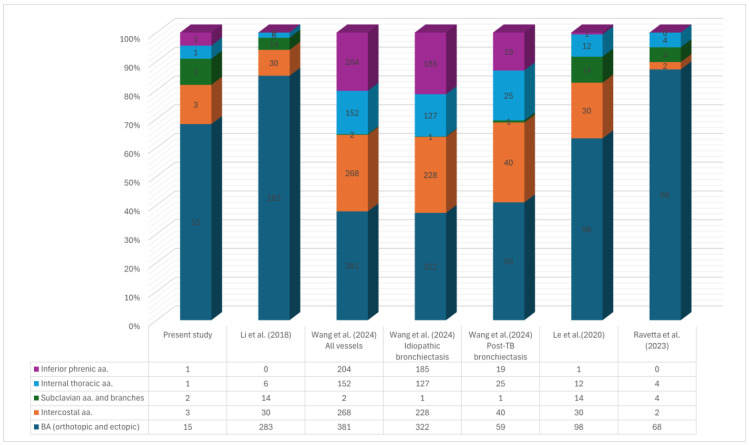
Numerical distribution of haemoptysis-related arteries: aggregated data from our study population and four prior series [11,12,23,24].

Aggregated data from studies reporting culprit vessel distribution revealed that orthotopic and ectopic BAs were most frequently identified as bleeding sources (Figure A1 and Figure A2). Among the analysed NBSAs, intercostal arteries were the most common, whereas subclavian arteries and their branches were the least frequent [11,12,23,24].

Although our study population was considerably smaller than that in comparative studies, the diversity of detected NBSAs was substantial. Notably, it included two cases of embolisation involving the subclavian artery, which has been reported in the literature as one of the rarest sources of haemoptysis.

An additional strength of our analysis is the long follow-up period after the first embolisation, particularly in chronically ill patients attending regular visits. In these patients, NBSAs were frequently the target of re-embolisation, which may reflect disease progression and chronic inflammation, as well as the development of collateral vessels following bronchial artery occlusion due to altered intrapulmonary pressure gradients. Progressive vascular and parenchymal remodelling in the lungs, including the formation of new collaterals, dilatation of pre-existing vessels and structural changes in the lung parenchyma, further supports the rationale for performing CTA before each embolisation. Moreover, the wide range of potential NBSA origins—from the subclavian to the phrenic artery underscores the necessity of conducting CTA with an extended imaging window.

In line with these observations, the recently published consensus statement by the Chinese College of Interventionalists reported complete agreement (100%) among experts on the need for enhanced CT/CTA in all patients with haemoptysis before AE, with a recommended imaging window extending from the neck to the level of the L2 vertebra (strong recommendation) [36]. Beyond the cranio-caudal extent, the transverse breadth of this imaging window is equally important, as it permits thorough visualisation of collateral vascular pathways—an observation corroborated by our own experience.

Herrera et al. demonstrated that performing a CT scan of the complete lung fields before AE does not significantly affect radiation dose, contrast volume or procedure duration, although their results showed a trend towards shorter procedure times when CTA was performed [37]. Li et al. suggested that MDCTA may reduce procedure time and radiation exposure [11]. Both sources agree on the value of CT in enabling procedural planning by the interventionist, for example, omitting some fluoroscopic steps or selecting the optimal vascular catheter, which may reduce overall exposure.

Herrera also suggested that CT is not necessary for effective treatment in patients with an already established underlying disease [37]. However, according to our observations, lung abnormalities in diseases associated with chronic inflammation change dynamically—opening of collaterals and neovascularisation occur continuously—making it difficult to predict the next bleeding source on the basis of earlier imaging. Under such conditions, the likelihood of missing an NBSA is high, which led Martin et al. to recommend that CTA should be performed within 6 months before AE [14]. Missing a feeding vessel is one of the more common causes of recurrent haemoptysis, and correct identification of bleeding sites may therefore reduce the need for re-embolisation, which also affects cumulative radiation exposure. This consideration is particularly important in young patients with CF, who often undergo repeated imaging studies. Nevertheless, in Herrera’s study, this effect was not demonstrated, and recurrences occurred in 36% of patients [37]. The study by Herrera did not account for the potential impact of the bleeding source—differences may have emerged if patients had been stratified according to BA or NBSA origin.

The wider imaging window proposed in our study may improve the detection of NBSAs at the cost of a slightly increased radiation dose compared with a standard lung-field protocol. The radiation dose in our study population fell within the expected diagnostic range and was adjusted individually according to patient body habitus. In our centre, we use iterative reconstruction protocols, which additionally reduce patient exposure while allowing high-quality imaging [38].

Radiation exposure associated with procedures performed for haemoptysis remains an area requiring further investigation, especially given the inconsistencies across available studies. An important aspect is balancing the radiation-related risk with the potentially life-threatening consequences of massive or recurrent haemoptysis. In the setting of massive haemoptysis, the immediate risk of asphyxiation and death necessitates urgent diagnostic and therapeutic intervention, and in this context, radiation-related concerns become secondary.

Both Li and Petel demonstrated that preprocedural CTA does not influence the technical success of AE, but it is valuable for assessing the underlying aetiology of haemoptysis and for the preliminary identification of culprit vessels. In Li’s study, it also improved the haemoptysis-free early survival rate. Importantly, in both of these studies, the predominant aetiology of haemoptysis was non-CF bronchiectasis, whereas in our study population, most patients had CF [11,39]. This condition makes vascular access more difficult due to complex anatomical alterations. The absence of a control group in our study precludes firm conclusions regarding the impact of CTA on technical success in the setting of markedly complicated pulmonary vascular anatomy. Nevertheless, further studies are warranted to determine the role of CTA in improving technical outcomes in CF patients, in whom AE is frequently performed, including as a bridge to lung transplantation [10,20,40].

In summary, this study highlights the variability of vascular supply in patients with life-threatening and recurrent haemoptysis and supports the need for detailed preprocedural imaging, including a wide plan, even in centres with limited case volumes. Although retrospective and descriptive in nature, our work underscores the clinical relevance of detailed preprocedural imaging and contributes to the growing body of evidence supporting CTA as an integral component of haemoptysis management.

## 5. Limitations

This study has several limitations. The main limitation is the small number of cases, which precludes generalisation and means that the findings should be considered exploratory and descriptive rather than confirmatory. Another limitation is the absence of a control group, which precludes direct comparison with alternative management strategies and limits the strength of causal inferences. The retrospective design may have introduced selection and information bias, while the single-centre setting limits the generalisability of the findings. Moreover, our department primarily focused on neurointerventional procedures and peripheral vascular malformations; operator experience in AE for haemoptysis was therefore limited, which may have influenced outcomes. In addition, the study population was heterogeneous with respect to clinical characteristics, which could have influenced treatment outcomes. This study did not account for the potential influence of different embolic agents or the severity of haemoptysis volume, both of which may have affected treatment outcomes. Finally, the duration of follow-up varied considerably among patients, potentially affecting the consistency of long-term observations.

## 6. Conclusions

In summary, our findings suggest that CTA performed from the neck to the L2 vertebral level is a valuable tool for AE planning in life-threatening haemoptysis, particularly in patients with complex vascular anatomy or progressive pulmonary changes.

By enabling the reliable identification of even rare systemic collateral vessels, such as phrenic or subclavian branches that are often overlooked in standard protocols, CTA may help reduce recurrence related to feeder omission. This approach may be crucial in pathologies that drive neovascularisation and engage NBSAs, especially in CF.

In three patients with CF, haemoptysis control was achieved when AE was combined with CFTR modulators, indicating potential complementary roles of interventional and medical therapy.

These observations are exploratory and limited by the small number of cases; nevertheless, they underscore the importance of performing preprocedural CTA from the neck to the L2 vertebral level in clinical practice.

## Figures and Tables

**Table 1:** Demographic and clinical characteristics of patients.

Case Number	Sex	Age at First Arterial Embolisation	Main Aetiology	Comorbidities	Number of Arterial Embolisation Procedures	Embolisation Material(s)	Follow-Up Outcome
P01	M	19	CF	CF-related diabetes; hypertension; asthma; allergic rhinitis	3	Coil Glue Other	Recurrent haemoptysis after three AEs; controlled with CFTR modulators; alive
P02	M	32	Aspergillosis	sarcoidosis	3	Glue	Effective AE; alive
P03	M	39	Post-TB sequelae	bronchiectasis	1	Coil	Lost to follow-up
P04	M	30	CF	Cholelithiasis	3	Coil Other	Lost to follow-up
P05	M	19	CF	Depression; anorexia	1	-	AE attempted, embolisation technically not feasible; alive
P06	F	62	Post-TB sequelae	Hypertension; renal cirrhosis	1	-	Lost to follow-up
P07	F	28	CF	Cholelithiasis; CF-related diabetes	1	Glue	Effective AE; on CFTR modulators; death
P08	F	33	Post-TB sequelae	Gastroesophageal reflux disease; anorexia	2	Glue	Effective AE; post-LTx; alive
P09	F	24	CF	CF-related diabetes; asthma	1	Other	Effective AE; on CFTR modulators; alive
P10	M	53	Pulmonary nontuberculous mycobacteriosis	Chronic obstructive pulmonary disease; ulcerative colitis; benign prostatic hyperplasia	1	Glue	Effective AE; listed for LTx; alive

Cystic fibrosis (CF), tuberculosis (TB), artery embolisation (AE), lung transplantation (LTx), cystic fibrosis transmembrane conductance regulator modulators (CFTR modulators), male (M), female (F).

**Table 2:** Characteristics of patients and embolisation procedures targeting NBSAs.

Case Number	Culprit Vessel Origin	Arterial Embolisation Procedure Number	Embolisation Agent
P01	Left subclavian artery	Third	Glue
P02	Right intercostal artery	Third	Coil
P03	Branch of the aorta at T12 (likely left inferior phrenic artery)	First	Coil
P04	Right subclavian artery	Third	Coil
P06	LIMA	AE was abandoned due to the severe tortuosity of LIMA	-
P08	Right and left intercostal artery	Second	Glue

Arterial embolisation (AE), left internal mammary artery (LIMA).

**Table 3:** Correlation between CTA findings and embolised culprit vessel.

Case Number	Computed Tomography Angiography Findings	Culprit Vessel Identified on Digital Subtraction Angiography	Match
P01	Dilated, tortuous R BA	R BA	Yes
	Dilated, tortuous R BA (up to 3 mm) Bilateral bronchiectasis (RUL, LUL, RML dominant) Mild consolidations	R BA	Yes
	Dilated, tortuous R BA (up to 2.9 mm) Bilateral bronchiectasis (RUL, LUL, RML dominant) Focal consolidation in apical segments (LUL)	L subclavian a.	Yes
P02	Consolidation in S2 (RUL)	R BA	Yes
	Progressive parenchymal changes in S5 and along the oblique fissure (RL)	R BA	Yes
	Consolidated changes at the lower hilar pole and in segment 6S R Pleural thickening up to 10 mm over segment 6S R	R intercostal a.	Yes
P03	LIMA dilated to 3.1 mm; L BA to 3.3 mm; L inferior phrenic a. to 3.1 mm Post-inflammatory changes bilaterally Fibrotic transformation of LL	L Th12 aortic branch (phrenic a.)	Yes
P04	Dilated, tortuous R BA (up to 5–6 mm) and L BA (up to 4 mm) Inflammatory changes within bronchiectatic regions supplied by R BA Fibrotic RML	R BA	Yes
	Dilated, tortuous R BA (up to 4–5 mm) and L BA (up to 4 mm) The right internal thoracic a. is involved in abnormal supply	2 × R BA	Yes
	Persistent vascular abnormalities, comparable to prior CTA	R subclavian a.	Yes
P05	Dilated, tortuous L BA (up to 2.6 mm) Pleural adhesions in S2 (RUL) and S4 (LUL)	R BA	No
P06	Consolidation in S6 of LLL Fibrotic right lung with basal fibrosis	LIMA	Yes
P07	Dilated, tortuous R BA (up to 4 mm) and L BA Diffuse GGO (especially in LLL)	2 × R BA	Yes
P08	Dilated multiple R BA (one from arch, two from descending aorta) (up to 3.2–3.6 mm) CT signs of fistulas/anastomoses in RUL Apical pleural thickening bilaterally	R BA	Yes
		R&L intercostal aa.	Yes
P09	Dilated R BA with suspected anastomosis to pulmonary artery, dilated L BA	R BA	Yes
P10	GGO in S3 of RUL	2 × R BA	Yes

Computed tomography angiography (CTA), computed tomography (CT), right (R), left (L), bronchial artery (BA), artery (a.), left internal mammary artery (LIMA), right upper lobe (RUL), left upper lobe (LUL), right middle lobe (RML), right lung (RL), left lung (LL), left lower lobe (LLL), ground-glass opacities (GGO), lung segment (S).

## Data Availability

The data presented in this study are available on request from the corresponding author. The data are not publicly available due to privacy and ethical restrictions.

## Abbreviations

The following abbreviations are used in this manuscript:

AE	Artery embolisation
CF	Cystic fibrosis
IMA	Internal mammary artery
CT	Computed tomography
ACR	American College of Radiology
CIRSE	Cardiovascular and Interventional Radiological Society of Europe
CTA	Computed tomography angiography
GGOs	Ground-glass opacities
NBSAs	Non-bronchial systemic arteries
MDCTA	Multidetector computed tomography angiography
DSA	Digital subtraction angiography
TB	Tuberculosis
L	Left
R	Right
BA	Bronchial artery
a.	Artery
aa.	Arteries
RUL	Right upper lobe
LUL	Left upper lobe
RML	Right middle lobe
RL	Right lung
LL	Left lung
LLL	Left lower lobe
S	Segment (Pulmonary segment)
N/A	Non-applicable

## Appendix A

**Table A1:** Demographic and clinical characteristics of patients with haemoptysis (our study population vs. Li et al. [11]). Both groups of embolised patients—those with and without CTA—were included for appropriate comparability between studies.

Patients		Present Study	Li et al. [11]
Number of patients		11	157
Age (years)	Mean age	36 ± 15	50 ± 14
	Min.	19	19
	Max.	62	79
Male/female ratio		7/4 (64%/36%)	113/44 (72%/28%)
Aetiology	CF	5 (45%)	N/A
	Tuberculosis	0 (0%)	27 (17%)
	Bronchiectasis (non-CF)	2 (18%)	76 (48%)
	Pulmonary embolism	1 (9%)	1 (1%)
	Aspergilloma	1 (9%)	3 (2%)
	Others	2 (18%)	50 (32%)
Embolic agent (per-procedure)	Glue	6 (33.3%)	0 (0%)
	Coil	6 (33.3%)	10 (6%)
	Polyvinyl alcohol	1 (5.6%)	123 (78%)
	Gelatin sponge	0 (0%)	0 (0%)
	Other	3 (16.7%)	24 (15%)

Cystic fibrosis (CF), non-applicable (N/A).
